# Emergency Clinical Decision for Sports Injury Management: A Wearable Sensor-Driven Framework from Training to Rehabilitation

**DOI:** 10.3390/bios16040205

**Published:** 2026-04-03

**Authors:** Maolin Xu, Shan Lang, Jichen Wang, Liang Huang, Meng Wang, Meng Su, Haiyan Zhu

**Affiliations:** 1 Department of Emergency, the First Medical Center, Chinese PLA General Hospital, Beijing 100853, China; dushubaoguo@163.com (M.X.); elmacho_wm@163.com (M.W.); 2 Department of Endocrinology and Metabolism, Peking University Third Hospital, Beijing 100191, China; langshan@bjmu.edu.cn; 3 Senior Department of Urology, Chinese PLA General Hospital, Beijing 100853, China; wangjichen301@163.com; 4 Senior Department of General Surgery, the First Medical Center, Chinese PLA General Hospital, Beijing 100853, China; hlsurgery@126.com; 5 Key Laboratory of Green Printing, CAS Research/Education Center for Excellence in Molecular Sciences, Beijing Engineering Research Center of Nanomaterials for Green Printing Technology, Beijing National Laboratory for Molecular Sciences (BNLMS), Institute of Chemistry, Chinese Academy of Sciences (ICCAS), Beijing 100190, China

**Keywords:** wearable sensors, sports injury, emergency medicine, injury management continuum, clinical decision support, rehabilitation monitoring, fatigue detection

## Abstract

Sports-related injuries present challenges across training, acute care, and rehabilitation, and largely rely on episodic, subjective, and delayed assessment methods. Wearable sensor technologies have emerged as powerful tools for objective monitoring of biomechanical and physiological parameters, offering new opportunities to enhance the entire sports injury management continuum. While prior research has explored the function for sports monitoring and injury prevention, the potential role of wearable sensors in the entire clinical pathway covering acute injury assessment, emergency clinical decision-making and rehabilitation guidance remains insufficiently integrated. This review synthesizes current advances in wearable sensor technologies, including inertial measurement units, pressure sensors, surface electromyography, cardiovascular monitoring, biochemical sweat sensing, and emerging self-powered and textile-integrated systems. Another main part of this review is the proposal of a wearable sensor–driven emergency clinical decision framework that integrates multimodal sensor data with clinically interpretable indicators to support risk assessment, early triage, treatment suggestions, and rehabilitation management. We also analyze the key challenges related to data integration and interpretation barriers, clinical implementation, ethical, privacy, and regulatory considerations. In the end, we look forward to the future of wearable sensors in data-driven, timely, and personalized sports injury care at the intersection of sports and emergency medicine.

## 1. Introduction

Sports-related injuries are a significant challenge to both athletic and general populations, and may lead to substantial physical, psychological, and socioeconomic burden. A report published in *British Journal of Sports Medicine* provides updated incidence data for elite athletes during major international competitions: for Paralympic athletes, approximately 14.3 injuries per 1000 Athlete-Days (AD); for Youth Olympic athletes, about 10.5 injuries per 1000 AD; and for Olympic athletes, 6.5 injuries per 1000 AD [1]. These figures reflect the injury risk among athletes with the highest levels. In adult recreational sports, injury incidence in golf has been estimated at 2.5 injuries per 1000 athlete exposures [2]. A meta-analysis indicates that hamstring injury incidence is 0.81 per 1000 h, representing 10% of all injuries in field-based team sports [3]. The assessment and management of sports injuries often rely on episodic clinical evaluations, subjective symptom reporting, and imaging-based diagnostics. Inappropriate diagnosis and treatment measures not only waste time but also affect the therapeutic outcome. A rapid, objective assessment is crucial but frequently unavailable in on-field, pre-hospital, or emergency department settings.

Wearable sensors have emerged as a promising solution to these challenges, enabling continuous, objective monitoring of biomechanical and physiological parameters. With advances in sensor miniaturization, wireless communication, and biocompatible materials, wearable devices are able to capture movement mechanics and physiological responses that were once limited to controlled laboratory environments. Over the past decade, wearable sensor systems have evolved from basic activity trackers to sophisticated, multimodal platforms integrating Inertial Measurement Units (IMUs), pressure-sensitive insoles, Surface Electromyography (sEMG), Heart Rate and Heart Rate Variability (HR/HRV) monitors, biochemical sweat sensors, and emerging self-powered technologies such as Triboelectric Nanogenerators (TENGs) [4,5]. These sensors can be positioned on the different body segments of athletes and monitor kinematic, kinetic, neuromuscular, cardiovascular, and biochemical signals. Wearable sensors have proven effective for injury prevention and training optimization by delivering quantitative data about workload, movement quality, and fatigue [6,7]. Similar technology is applied in rehabilitation, enabling clinicians to track functional recovery objectively, guide biofeedback-driven exercises, and make better-informed return-to-sport judgments [8].

While many wearable sensor systems demonstrate advanced technical performance, only a small proportion of them are ready for real-world clinical deployment, especially in time-sensitive, resource-constrained emergency environments [9]. Current research has mainly emphasized sensor validation and algorithm development. Relatively little attention is paid to how sensor-derived data can inform emergency triage, early risk warning, and immediate management decisions in sports-related injuries. And studies largely examine isolated segments of the injury lifecycle, leaving a notable gap in integrated strategies that bridge injury prevention, acute care, and rehabilitation guidance. The role of wearable sensors in the entire management continuum of acute injury detection, emergency clinical decision-making, and rehabilitation has not been adequately explored so far. Advances in Artificial Intelligence (AI), including Machine Learning (ML), have the potential to bridge this gap. AI-driven approaches can transform complex, high-dimensional sensor data into clinically interpretable outputs by enabling automated feature extraction, injury classification, and multimodal data fusion [10,11]. At the same time, innovations in smart materials, textile-integrated sensors, and self-powered systems are expected to address long-standing challenges related to battery life, comfort, and long-term wearability, which are critical for continuous monitoring during the entire process [5,12].

Yet no unified framework exists for integrating wearables into emergency sports injury assessment while preserving continuity with warning reminder, prevention and rehabilitation. Emergency physicians often lack objective, real-time data during the acute injury phase, making rapid triage difficult—particularly when distinguishing minor injuries from those demanding urgent imaging, specialist referral, or activity restriction. This review synthesizes evidence on wearable sensors throughout the entire sports injury management continuum, emphasizing acute injury detection, emergency assessment, clinical decision support, and rehabilitation guidance. We integrate biomechanical, neuromuscular, physiological, and biochemical sensing to propose a sensor-driven framework for emergency decision-making, embedding these technologies within larger training and rehabilitation workflows. Wearable sensors are not simply monitoring tools, but also as drivers of timely, personalized, data-informed injury management across sports and emergency medicine.

## 2. The Widely Applied Wearable Sensor Technologies

**Figure 1:**
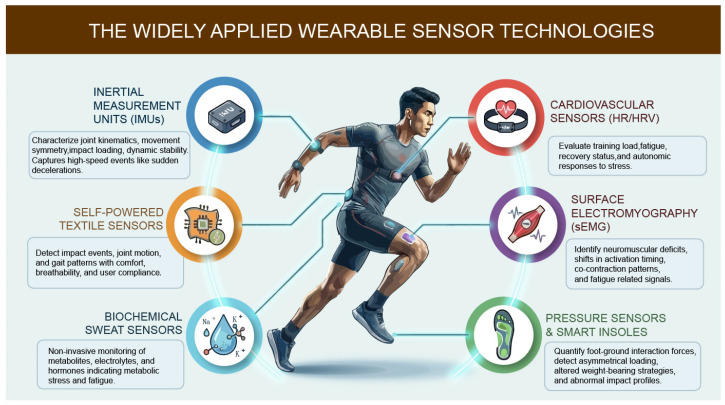
The widely applied wearable sensors and their specific applications.

Wearable sensor technologies serve as the base of objective, real-time evaluations of sports. Unlike conventional laboratory-based motion analysis systems, wearable sensors enable continuous tracking of biomechanical and physiological parameters under real-world scenarios—including training sessions, competitive events, and clinical settings. Such technologies have particular value for sports injury assessment, as they can capture both gradual changes that precede injury and acute shifts linked to injury onset and recovery [9]. To date, wearable sensor systems can be classified based on the type of information they gather, such as biomechanical motion, plantar loading, neuromuscular activity, cardiovascular responses, and biochemical status. Each sensor modality offers distinct insights into injury mechanisms, severity, and recovery trajectories. The widely applied wearable sensors and their specific applications are presented in Figure 1. Section 2.1, Section 2.2, Section 2.3, Section 2.4 and Section 2.5 focus on core sensing modalities categorized by their intrinsic detection principles and measured biological parameters (Table 1), while Section 2.6 presents system-level integration innovations and translational application paradigms (e.g., self-powered, textile-integrated designs) that enhance the real-world performance and usability of these core sensing modalities.

### 2.1. Inertial Measurement Units

IMUs are among the most widely used wearable sensor technologies in sports injury research, accounting for the majority of published studies [9]. A common IMU brings together accelerometers, gyroscopes, and, in select configurations, magnetometers to quantify linear acceleration, angular velocity, and segment orientation across Three-Dimensional (3D) space. IMUs excel at capturing transient, high-speed events, such as sudden decelerations, cutting maneuvers, or landing impacts that prove challenging to evaluate through traditional clinical examinations alone [13]. For sports injury assessment, IMUs are primarily employed to characterize joint kinematics, movement symmetry, impact loading, and dynamic stability [14]. These aspects are directly tied to common injury mechanisms, including non-contact ligament injuries, overuse injuries, and episodes of joint instability. A case study about ankle “giving way” during landing tasks demonstrated that IMUs could capture characteristic patterns of increased inversion (55° vs. 42° in normal trials) and internal rotation that preceded instability events [15]. However, IMU measurements may be affected by Motion Artifacts (MAs) and sensor displacement during high-impact movements, and accurate orientation estimation often requires careful calibration.

### 2.2. Pressure Sensors and Smart Insoles

Plantar pressure sensors and smart insoles offer a direct method for quantifying foot-ground interaction forces, giving unique insights into lower-limb loading patterns [16]. These systems typically employ capacitive, resistive, and piezoelectric sensor arrays on the plantar surface to measure pressure magnitude, distribution, and temporal characteristics during gait and sport-specific movements. When it comes to injury assessment, pressure sensors stand out for their utility in detecting asymmetrical loading, altered weight-bearing strategies, and abnormal impact profiles following injury [17]. Such changes are common after acute lower-extremity injuries and often escape easy detection during static clinical examinations [18]. Supporting comes from a prospective double-blind study, which found that a Shoe-Integrated Sensor System (SISS) could detect associated syndesmotic injuries while walking, achieving 80% sensitivity and 75% specificity [19]. In another trial, participants who got real-time feedback from wearable insoles experienced significantly fewer injuries (HR = 0.53; *p* = 0.03) [20]. Technical advances continue to improve these tools. Wu et al. [21] developed capacitive pressure sensor arrays for a lower limb motion capture system, featuring an ultra-wide pressure range (up to 3770.9 kPa), high sensitivity (2.68 kPa^−1^), and rapid response time (17.2 ms). Lee et al. [22] fabricated 173 carbon-based sensors directly onto a flexible insole circuit via a screen-printing method, demonstrating a remarkable sensitivity of −0.322 kPa^−1^. These characteristics enable the detection of subtle gait abnormalities and loading patterns that may be beneficial for indicating injury risk or tracking rehabilitation progress. Nonetheless, plantar pressure sensors may experience signal drift under repetitive high loading, and sensor alignment can directly influence the accuracy of regional pressure distribution measurements.

### 2.3. Surface Electromyography

SEMG sensors detect the electrical signals generated by skeletal muscle contractions, offering direct insight into the activation patterns, temporal dynamics, and coordination mechanisms of the neuromuscular system. In the context of sports injury evaluation, sEMG is uniquely valuable for pinpointing neuromuscular impairments—these may either persist as sequelae of prior injuries or arise as compensatory motor strategies during functional movements. Wearable sEMG devices usually integrate dry or gel-based electrodes into flexible materials, enabling real-time signal collection during dynamic exercises that extend beyond the constraints of laboratory environments. Alterations in muscle activation timing, patterns of muscular co-contraction, and signal features linked to muscle fatigue have usually been correlated in endurance sports and the prolonged recovery process [10]. Despite these advantages, sEMG signals are susceptible to electrical noise, crosstalk, and MAs during dynamic movements, and electrode placement significantly affects signal consistency.

Wearable sensing devices constructed on hydrogel substrates deliver outstanding efficacy for non-invasive electrophysiological monitoring, extending across Electrocardiography (ECG), Electromyography (EMG), and Electroencephalography (EEG) applications. The key advantages include high signal fidelity, stretchability up to 1879% strain, and long-term stability, support in continuous recording for 8+ days with ultra-thin (10 μm) breathable films, reducing blink artifacts [23].

Recent advances that combine sEMG with IMU data and ML algorithms have improved the interpretability of neuromuscular signals [24,25]. Bai et al. [10] integrated sEMG with IMU data through advanced algorithms such as the SWCTNet (Sliding Window CNN + Channel-Time Attention Transformer Network), which enables the prediction of muscle activation patterns from motion data alone. This approach has demonstrated classification accuracies ranging from 87.93% to 91.03% on public datasets and 98% on self-collected datasets, providing a possibility for simplified field-based assessment of neuromuscular function.

### 2.4. Cardiovascular Sensors: Heart Rate and Heart Rate Variability

Wearable cardiovascular sensors, such as HR and HRV monitors, deliver key points into autonomic nervous system function and systemic physiological stress. Modern wearable systems employ Photoplethysmography (PPG) or ECG-based approaches, which are well-suited to capturing beat-to-beat variability across varying sampling rates [26,27]. For sports injury assessment, HR and HRV metrics are widely used to evaluate training load, fatigue, and recovery status. Growing evidence indicates that altered autonomic responses may also reflect acute stress reactions following injury, and potentially influence early rehabilitation [28]. Bahenský et al. [6] demonstrated that adolescent runners using HRV-guided training at altitude had better adaptations compared to conventional approaches, with 4.27% improvements in VO_2_max and reduced injury rates.

On the other hand, HR and HRV measurements can be influenced by MAs, environmental stressors, and individual physiological differences, which may reduce their specificity for injury-related stress evaluation.

### 2.5. Biochemical Sweat Sensors

Biochemical sweat sensors are an emerging type of wearable technology, enabling non-invasive assessment of metabolites, electrolytes, and hormones associated with physical stress and tissue damage. These devices typically adopt electrochemical or colorimetric detection methods to quantify essential biomarkers like lactate, glucose, sodium, and cortisol [29,30,31,32,33]. For sports injury assessment, sweat biomarkers offer indirect indicators of metabolic stress, fatigue, and systemic inflammatory responses. While most applications remain exploratory, advances in sensor stability and multianalyte detection point to potential roles in monitoring physiological responses to injury and recovery—particularly during prolonged rehabilitation or high training loads [34]. A review by Singh et al. [33] introduced the properties of graphene and its derivative-based wearable sensors for the detection of analytes in sweat. Konno et al. [30] reported that a wristwatch sweat lactic acid monitor can detect lactic acid distribution at the skin surface during stationary bike exercise. Su et al. [35] demonstrated that hypoxanthine concentration changes in sweat served as a reliable biomarker for diagnosing exercise-induced fatigue, and shifts in sweat pyruvate content can distinguish the body’s energy metabolism modes both pre- and post-exercise. Nevertheless, current sweat-based biochemical sensors still face challenges, including limited long-term stability, variable repeatability, and potential interference from skin temperature and sweat rate.

### 2.6. Self-Powered and Textile-Integrated Sensors

This section presents integrated system-level advancements and practical applications for the core sensing modalities described earlier. Self-powered wearable sensors—particularly TENGs—resolve key limitations of battery life and continuous monitoring [36]. TENG-based sensors harvest mechanical energy from human motion to generate electrical signals, enabling long-term operation without external power sources [5,37]. With high sensitivity, rapid response times, and excellent durability, these systems are well-suited for detecting impact events, joint motion, and sports injury-associated gait patterns. Meanwhile, textile-integrated sensors embed sensing elements directly into garments, enhancing comfort, breathability, and user compliance during prolonged wear [12].

Emerging wearable sensor technologies are evolving rapidly, such as wearable optical devices [38], microfluidic-integrated systems [32], 3D-printed platforms [37,39], and nanoscale/microscale detectors [40]. Additionally, research on the application of smart materials—self-healing compounds, metamaterials, responsive substances, and biodegradable polymers [41]—continues to deliver promising breakthroughs.

## 3. Data Processing and Multimodal Integration Strategies for Clinical Translation

Sport-focused wearable sensors differ fundamentally from daily-use consumer wearables. Unlike devices designed for low-intensity, steady-state activities such as walking or resting, sport-specific sensors are engineered to withstand high-speed, high-impact, and transient dynamic movements encountered during athletic training and competition [42]. They feature wider measurement ranges, higher sampling rates, and improved resistance to MAs, enabling reliable capture of biomechanical and physiological signals related to movement quality, loading symmetry, dynamic stability, and injury-relevant abnormalities [43]. Furthermore, sport-targeted sensors emphasize durability, sweat resistance, and secure attachment to support prolonged use in realistic training and competitive environments [44]. These design principles distinguish sport-specific sensors and underpin their unique value for performance analysis, injury prevention, and acute injury assessment.

**Figure 2:**
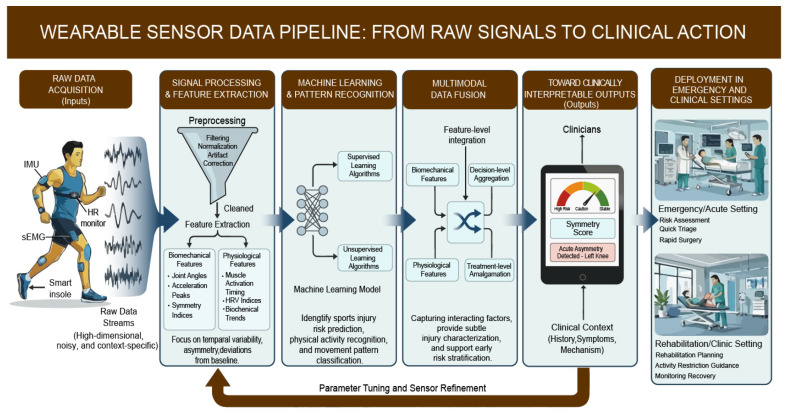
The wearable sensor data pipeline: from raw signals to clinical action.

Raw data generated by wearable sensors are typically high-dimensional, noisy, and context-specific, that restrict their direct application in clinical and emergency environments [9]. To bridge the gap between sensor data collection and clinical decision-making, data processing workflows usually need to integrate signal preprocessing, feature extraction, pattern recognition, and multimodal data fusion. Each of these steps is critical for converting continuous sensor outputs into interpretable metrics in medicine. Figure 2 shows the wearable sensor data pipeline: from raw signals to clinical action.

### 3.1. Signal Processing and Feature Extraction

Wearable sensor data are highly susceptible to MAs, environmental interference, and inter-individual variability. For this reason, preprocessing serves as a critical step in preserving data quality and reliability. Common preprocessing approaches include noise-filtering to remove extraneous signal interference, signal normalization to account for variability across sessions or subjects, and artifact correction to mitigate effects from sensor displacement or soft tissue motion [45].

Signal filtration, a core preprocessing step for wearable sensor signals, isolates target physiological signals from interference (e.g., MAs, power-line noise, ambient light fluctuations) by distinguishing signal and noise frequency bands. Its fundamental mechanism involves selecting specific frequency ranges: low-pass, high-pass, band-pass, and notch filters—typically designed via Finite Impulse Response (FIR) or Infinite Impulse Response (IIR) methods—effectively suppress unwanted frequencies [46]. Advanced adaptive filtering (e.g., Least Mean Squares (LMS) filters) further optimizes performance by dynamically adjusting coefficients to adapt to rapidly changing noise profiles in wearable scenarios, enabling real-time noise removal without distorting biological data.

Recent progress extends beyond basic filtration. Time-frequency analysis techniques (Short-Time Fourier Transform (STFT), Wavelet Transform, Empirical Mode Decomposition (EMD)) address non-stationary biomedical signals (e.g., ECG, EMG, PPG) [47,48]. Wavelet Transform excels in time-frequency feature localization, while EMD adaptively decomposes signals into intrinsic mode functions. Additionally, data compression techniques (Huffman coding, Run-Length Encoding (RLE), compressed sensing) have advanced to mitigate wearable device constraints (power, bandwidth, storage), enabling efficient high-frequency data transmission and long-term monitoring.

Following preprocessing, feature extraction is employed to distill raw sensor data into meaningful parameters that capture key aspects of movement and physiological function. For biomechanical sensors like IMUs and pressure insoles, extracted features might include joint angle ranges, acceleration peaks, symmetry indices, loading rates, and gait parameters. For physiological sensors, features can include muscle activation timing from sEMG, HRV indices, or variation trends in biochemical markers [49]. Al-Sheikh [50] reported the use of the Discrete Wavelet Transform–Recursive Inverse (DWT-RI) adaptive filter algorithm and Multiresolution Analysis (MRA) to denoise raw PPG signals, suppress MAs, and estimate HR, resulting in an absolute average error of 1.17 beats/min when tested on 12 subjects from a database.

Notably, many sport injury-related changes are subtle and may not be fully reflected in absolute values alone. Temporal variability, interlimb asymmetry, and deviations from an individual’s baseline yield more clinically relevant insights than population-level reference norms.

### 3.2. Machine Learning and Pattern Recognition

Given the multivariate and nonlinear characteristics of wearable sensor data, ML techniques have become increasingly important for uncovering sport injury-related patterns that may be difficult to detect using conventional statistical methods [51]. Jeong et al. [52] summarized the key algorithms and application workflows of ML in biosignal analysis for wearable devices, covering core methods including data preprocessing, clustering, regression, and classification, discussing recent advances in multimodal signal monitoring and disease diagnosis across neural, cardiovascular, and biochemical domains. Supervised learning algorithms are commonly used to classify movement patterns, detect abnormal biomechanics, or estimate injury severity based on labeled datasets, while unsupervised approaches identify latent patterns and deviations without predefined injury labels [11]. Within sports injury research and applications, tree-based models, Convolutional Neural Networks (CNNs), and recurrent neural networks have all proven effectiveness in core tasks—including sports injury risk prediction, physical activity recognition, and movement pattern classification [10]. Loke et al. [53] reported a fabric-based device, using a well-trained CNN model, which can accurately recognize human activities with an average accuracy of 96.4%. Karnuta et al. [54] tested 6 algorithms to develop 84 distinct models, which achieved predictive accuracies of 70% and 94.6% for clinical outcomes of sports injuries in baseball and hockey players, respectively. Another notable example is the combination of sEMG, IMU data, and ML algorithms mentioned earlier. These models process time-series data effectively and capture the complex interplay between biomechanical and physiological signals.

### 3.3. Multimodal Data Fusion

Sports injuries typically involve complex interactions between biomechanical loading, neuromuscular control, and physiological stress. For this reason, single-sensor strategies offer limited insight into injury mechanisms. Multimodal data fusion integrates information from multiple sensor types—such as IMUs, sEMG, pressure sensors, and cardiovascular monitors—to provide a more comprehensive and accurate assessment. The fusion can be implemented at various levels, including feature-level integration, decision-level aggregation, and treatment-level amalgamation. For example, integration of IMU and sEMG data via hybrid neural network architectures has achieved 98% accuracy in movement patterns classification [10].

From a clinical perspective, multimodal integration is especially valuable for acute injury assessment because biomechanical abnormalities may coexist with neuromuscular inhibition or increased physiological stress. By capturing these interacting factors, multimodal systems are better equipped to provide subtle injury characterization and support early risk stratification.

### 3.4. Toward Clinically Interpretable Outputs

A major barrier to the clinical adoption of wearable sensor technologies is the challenge of translating complex analytical outputs into actionable information that clinicians can easily interpret and apply. Importantly, those outputs should be designed to support rapid interpretation rather than exhaustive data review, aligning with the time-sensitive demands of emergency care. Decision-support systems that combine wearable sensor data with clinical context (e.g., injury mechanisms, symptom severity, and prior injury history) are essential for ensuring real-world utility. The primary goal of these systems is to enhance clinical judgment: they deliver objective data to guide decisions on imaging, activity restriction, patient referrals, and rehabilitation planning. For example, physicians can use remotely wearable devices to monitor cardiac health—including abnormal ECGs and recordings of arrhythmias or palpitations—in youth, collegiate, and professional athletes recovering from COVID-19 (Coronavirus Disease 2019) infection [7].

### 3.5. Deployment in Emergency and Clinical Settings

Despite some challenges, wearable sensor analytics show great potential for deployment in emergency and clinical settings. As technology continues to improve, sensor accuracy, placement variability, and data quality issues will be increasingly mitigated. Advances in data processing and multimodal integration have enabled real-time, objective assessment of injury severity, functional impairment, and physiological responses. Furthermore, expanding clinical datasets and standardizing sensor-derived metrics will enhance the reliability and application of these systems in clinical settings. When traditional diagnostic tools may be delayed or unavailable, wearable sensors can deliver valuable data. As systems become more streamlined and validated, they will seamlessly integrate into clinical workflows, providing faster and more informed injury management. In emergency and acute settings, risk assessment, quick triage, and rapid surgery advice can be made with the help of wearable sensors integration. In the clinic and rehabilitation setting, embedded wearable sensors are beneficial to rehabilitation planning, activity restriction guidance and monitoring recovery. Preatoni et al. [9] reported that 12% (20/165) of the studies examined achieved field-based deployment status, while the remainder were restricted to either the research or development level. In a prospective longitudinal cohort study, Ransom et al. [55] enrolled high school student athletes equipped with a Fitbit Sense for 4–6 weeks after injury clearance. For the orthopedic injury cohort, individual adherence rates ranged from 82% to 100%, whereas the concussion cohort 37% to 100%. The satisfactory adherence rates may indicate the possibility of wearable sensors deployed in clinical practice.

## 4. Wearable Sensors Across the Sports Injury Management Continuum

Sports-related injuries should be considered as dynamic processes rather than isolated incidents. Injury risk accumulates over time, often influenced by training loads, and is manifested through specific biomechanical or physiological disruptions at the moment of injury. The recovery process and return to sport further evolve as part of the injury. Wearable sensors are uniquely positioned to capture this continuum, offering continuous, objective measurements at each stage—from injury prevention during training to acute injury detection and emergency assessment, and through rehabilitation and return-to-sport decisions. This section summarizes the current evidence on how wearable sensors contribute to injury prevention, acute injury detection, emergency assessment, and rehabilitation monitoring, integrated within a unified framework.

### 4.1. Training Monitoring and Injury Prevention

During the pre-injury phase, wearable sensors are extensively used to monitor training load, movement quality, and fatigue-related changes that may increase the risk of injury. IMUs and pressure sensors are useful for quantifying external workloads, impact exposure, and movement patterns during sport-specific activities. Metrics derived from related sensors—such as cumulative loading, acceleration profiles, and asymmetry indices—provide objective indicators of mechanical stress, which are often difficult to assess through subjective reporting alone [7]. Physiological monitoring via HRV and sweat-based biochemical sensors further supplements biomechanical data by capturing systemic responses to training-induced stress. Shifts in autonomic balance or metabolic markers may signal inadequate recovery or heightened injury risk, underscoring the importance of integrating both physiological and biomechanical datasets into injury prevention frameworks [6,29].

### 4.2. Acute Sports Injury Detection and Emergency Assessment

Even with preventive measures in place, wearable sensors deliver critical data during the acute phase of injuries—when rapid, objective assessment is non-negotiable. In this context, “injury detection” does not refer to direct diagnosis of structural tissue damage, but rather the identification of acute biomechanical, neuromuscular, and physiological abnormalities indicative of injury onset and functional impairment. Sudden shifts in biomechanical signals, such as abnormal joint motion, abrupt decelerations, or altered loading patterns, can flag the occurrence of injury-related events and enable real-time characterization of injury mechanisms.

From an emergency medicine standpoint, wearable sensors have the potential to support early triage and risk stratification. Objective data on movement capacity, neuromuscular activation, and physiological stress can aid in distinguishing between minor injuries and those that require urgent imaging, specialist referrals, or activity restriction. Importantly, the information can be collected in on-field, pre-hospital, or emergency department settings—environments where traditional diagnostic resources may be delayed or limited.

For example, IMUs positioned near vulnerable joints are capable of detecting transient instability events, excessive joint movement, or atypical rotational patterns linked to ligament injuries [15]. Similarly, pressure-sensitive insoles can identify immediate changes in weight-bearing behavior following lower-limb injuries. This provides objective evidence of functional impairment, even when pain reports are unreliable or delayed [18]. In the chaotic and time-sensitive context of acute injury and emergency assessment, self-powered and textile-integrated sensors further improve the translational feasibility of wearable monitoring. They enable uninterrupted monitoring without the need for battery charging or complex setup procedures [5].

### 4.3. Rehabilitation Monitoring and Return-to-Sport Evaluation

Following initial injury management, wearable sensors grow increasingly valuable for monitoring rehabilitation progress and informing return-to-sport decisions. Traditional rehabilitation assessments typically depend on periodic clinical evaluations—an approach that often overlooks transient or task-specific deficits. Wearable sensors, by contrast, enable continuous tracking of functional recovery during real-world activities.

IMUs and pressure sensors are widely used to evaluate the restoration of joint kinematics, movement symmetry, and loading tolerance during rehabilitation exercises and sport-specific tasks [8]. Pressure sensors monitor weight-bearing patterns and loading rates during rehabilitation activities, enabling precise titration of loading to optimize tissue healing. Song et al. [56] report that instrumented insoles have demonstrated the ability to detect changes in Achilles tendon loading that correlate with plantar flexor function (r = 0.687) in patients with Achilles tendinopathy. (sEMG) adds further value by identifying persistent neuromuscular inhibition or altered activation patterns—issues that could raise reinjury risk if not addressed [10]. Wearable sensors also facilitate biofeedback-guided rehabilitation, delivering real-time input on movement quality and exercise execution. Compared to conventional rehabilitation methods, such systems have been shown to improve adherence, movement precision, and functional outcomes [57]. Full-textile gait recognition systems integrated with multipoint body sensing networks enable remote guidance of rehabilitation training via ML-based analysis of periodic signals and dynamic parameters [12]. Additionally, cardiovascular and biochemical monitoring provides complementary data on physiological readiness, supporting clinicians in tailoring rehabilitation intensity and progression.

Perhaps most critically, wearable sensors offer a more objective and personalized framework for return-to-sport decision-making. By integrating biomechanical performance, neuromuscular control, and physiological recovery markers, sensor-based assessments can reduce dependence on time-based criteria and subjective judgments. This, in turn, helps lower reinjury risk during the transition back to full sports participation.

### 4.4. Wearable Sensor-Driven Emergency Clinical Decision Framework

Wearable sensors offer a valuable opportunity to enhance clinical decision-making by providing real-time, objective data acquisition and analysis. One of the most promising uses of wearable sensors in emergency medicine is injury triage. Objective metrics of functional impairment can assist in early categorization of injury severity, shaping decisions around imaging, referrals, and activity restriction. These advantages may merge with the Three-Zone and Four-Level triage system in the emergency medicine department, refreshing the face of emergency treatment for sports injuries.

For example, in sports injuries, athletes whose HR, HRV and electroencephalogram monitors indicate sudden death are at Level 1 (immediate life-threatening patients) and should come to the Red Zone (resuscitation area) for immediate rescue without a routine electrocardiogram examination. In endurance sports such as Marathons and Triathlons, when biochemical sweat sensors detect emergency changes in metabolic components, extensive muscle contusion combined with Rhabdomyolysis may occur, which may further lead to acute renal failure. The athlete was classified as level 2 (emergent patients) and also promptly sent to the Red Zone for first aid. Fractures are warned by IMUs in competitive sports such as football and basketball can be defined as level 3 (urgent patients) and enter the Yellow Zone (emergency treatment area) for treatment before the imaging diagnosis. A mild lateral ankle ligament strain discovered by smart insoles is Level 4 (non-urgent patients) waiting in the green zone (waiting area) with no need to worry or be overly anxious.

In the subsequent treatment, for example, persistent asymmetry in joint motion or loading identified right after an injury may point to structural damage—even when clinical signs are not immediately obvious. On the other hand, near-normal functional measures despite reported pain might indicate that conservative management with close follow-up is suitable. The integration of parameters from wearable sensors and electronic health records or mobile clinical platforms can further enhance the intelligent diagnosis mode [57].

One of the key advantages of wearable sensors is their ability to provide continuity throughout the entire injury lifecycle, from prevention to rehabilitation. Rather than treating each phase as a separate clinical segment, wearable sensor data allow for longitudinal tracking of an individual’s baseline function, deviations related to injury, and progress during recovery. This continuity is particularly valuable in emergency and sports medicine contexts. Baseline data collected during training can offer crucial context for interpreting acute injury measurements, while post-injury data can be compared to pre-injury norms rather than broad population averages. These personalized reference points improve the precision of clinical assessments and enable more tailored management strategies.

**Figure 3:**
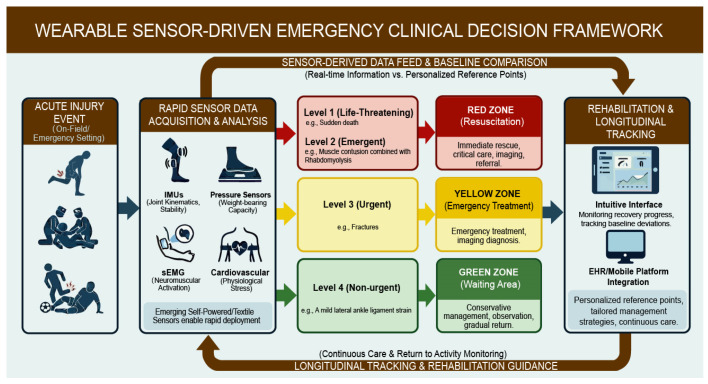
**Schematic diagram of a wearable** sensor-driven emergency clinical decision framework.

In this review, we propose a wearable sensor–driven emergency clinical decision framework designed not to replace clinical judgment, but to complement it. This framework provides a clear process including risk assessment, early triage, treatment suggestions, and rehabilitation management driven by a wearable sensor (Figure 3). It integrates sensor data acquisition, multimodal data fusion, and clinically interpretable outputs into existing emergency workflows.

## 5. Challenges and Translational Barriers

Despite the rapid progress in wearable sensor technologies and the growing body of evidence supporting their use, several challenges hinder their widespread adoption in clinical and emergency settings. These challenges are not confined to hardware limitations but span issues related to technical performance, data interpretation, clinical integration, as well as ethical and regulatory concerns. Overcoming these barriers is crucial for translating wearable sensor-driven approaches from research into clinical and emergency practice [9].

### 5.1. Data Integration and Interpretation Barriers

Wearable sensor systems generate heterogeneous data with various sampling rates, formats, and latencies. Integrating these data streams into unified, time-aligned representations remains technically challenging, particularly in multimodal systems used for emergency use [13]. Another major barrier to clinical translation is the absence of standardized, clinically validated thresholds for many sensor-derived metrics. Although relative changes and asymmetry indices are commonly reported, their direct implications for diagnosis, triage, or management decisions remain poorly defined [11]. This gap is especially critical in emergency settings, where actionable thresholds are required for rapid decision-making.

ML models trained on narrow or homogeneous datasets may not generalize well across different sports, populations, or injury types. Furthermore, complex algorithmic models may function as “black boxes,” reducing clinician trust and hindering adoption. For wearable sensors to gain clinical traction, explainable and robust analytical approaches are therefore essential [10].

### 5.2. Clinical Implementation Challenges

Clinicians lack formal training in interpreting wearable sensor data, particularly when outputs are presented as abstract metrics or probabilistic scores. Without user-friendly interfaces and clear clinical relevance, those systems risk being perceived as burdens rather than aids. User-centered design and clinician involvement in system development are critical for improving acceptance and sustained use [9].

Most studies of wearable sensors have been conducted in controlled laboratory settings or among healthy athletic populations. There is limited evidence supporting the use of these systems in injured patients, across diverse age groups, or in emergency department settings. Robust, prospective studies that evaluate the clinical impact—rather than just technical performance—are urgently needed to support broader adoption.

### 5.3. Ethical, Privacy, and Regulatory Considerations

Wearable sensors collect continuous, highly personal health data, raising concerns regarding data ownership, access, and secondary use. In sports contexts, competing interests among athletes, teams, and healthcare providers further complicate data governance. Transparent policies regarding consent, data use, and storage are essential to maintain trust and protect patient autonomy.

The regulatory status of wearable sensors used for injury assessment remains ambiguous, particularly at the intersection of consumer devices and medical technologies. Inconsistent regulatory pathways and validation requirements across jurisdictions may hinder clinical deployment and innovation. The use of sensor-derived data in clinical decision-making raises questions regarding liability, particularly when decisions are influenced by algorithmic outputs. Clarifying the role of wearable sensors as decision-support tools—rather than autonomous decision-makers—is critical to mitigating legal and ethical concerns.

Collectively, these challenges highlight that the primary barriers to wearable sensor adoption in emergency sports injury assessment are not purely technological but also systemic. Bridging these gaps will require coordinated efforts to standardize validation protocols, develop clinically meaningful metrics, integrate sensor systems into existing workflows, and establish clear ethical and regulatory frameworks.

## 6. Conclusions and Future Perspectives

Wearable sensor technologies have developed rapidly and offer unique opportunities to quantify biomechanical, neuromuscular, physiological, and biochemical aspects of sports-related injuries. As mentioned in this review, these technologies enable objective assessment across the entire sports injury management continuum—from injury prevention during training to acute injury detection and emergency assessment, and throughout rehabilitation and return-to-sport decision-making. The shift from episodic, subjective evaluation to data-driven, longitudinal injury management represents a fundamental advancement in this field.

Moving forward, the clinical impact of wearable sensors will hinge not merely on advancements in data volume or algorithmic complexity, but on how meaningfully they integrate into real-world clinical workflows. Emerging innovations in multimodal data fusion, explainable AI, self-powered sensing platforms, and textile-integrated systems provide promising paths to boost the feasibility, usability, and clinician acceptance of these technologies. Additionally, developing standardized metrics, conducting robust validation in emergency and injured populations, and establishing clear ethical and regulatory frameworks will be vital to ensuring responsible clinical deployment.

Ultimately, wearable sensors should be viewed as integral components of an interconnected clinical ecosystem—one that bridges training environments, emergency care, and rehabilitation pathways. When implemented thoughtfully, wearable sensor-driven strategies can improve diagnostic precision, speed up clinical decision-making, and support more personalized, timely injury management. Interdisciplinary collaboration between engineers, clinicians, and data scientists will be key to translating technological progress into tangible improvements in sports and emergency medicine practice.

## Figures and Tables

**Table 1:** Key Features of Different Wearable Sensor Modalities for Sports Injury Monitoring.

No.	Sensor Modality	Core Measured Parameters	Primary Applications in Sports Injury Monitoring	Key Advantages	Main Limitations
1	Inertial Measurement Units (IMUs)	3D linear acceleration, angular velocity, segment orientation	Characterize joint kinematics, movement symmetry, impact loading, dynamic stability; capture transient high-speed events	Widely used; excel at capturing high-speed events difficult to assess clinically	Susceptible to Motion Artifacts (MAs) and sensor displacement; requires calibration
2	Pressure Sensors and Smart Insoles	Plantar pressure magnitude, distribution, temporal characteristics	Detect asymmetrical loading, altered weight-bearing, abnormal impact, and subtle gait abnormalities	Quantify foot–ground interaction; detect changes missed in static exams	Prone to signal drift under repeated loading; sensitive to alignment
3	Surface Electromyography (sEMG)	Muscle electrical activity, activation timing, co-contraction, fatigue features	Identify neuromuscular impairments and compensatory strategies post-injury	Non-invasive real-time monitoring; high signal fidelity and stretchability	Affected by noise, crosstalk, and MAs; sensitive to electrode placement
4	Cardiovascular Sensors (HR & HRV)	HR, HRV, beat-to-beat fluctuations	Evaluate training load, fatigue, recovery, and post-injury physiological stress	Reflect autonomic nervous function; suitable for continuous monitoring	Influenced by MAs, environment, and individual variability
5	Biochemical Sweat Sensors	Lactate, glucose, sodium, cortisol, hypoxanthine, pyruvate	Assess metabolic stress, fatigue, inflammation, and rehabilitation status	Non-invasive biochemical monitoring; suitable for prolonged assessment	Limited stability and repeatability; affected by sweat rate and skin conditions

## Data Availability

No new data were created or analyzed in this study. Data sharing is not applicable to this article.

## Abbreviations

The following abbreviations are used in this manuscript:

AD	Athlete-Days
IMUs	Inertial Measurement Units
sEMG	Surface Electromyography
HR	Heart Rate
HRV	Heart Rate Variability
TENGs	Triboelectric Nanogenerators
AI	Artificial Intelligence
ML	Machine Learning
SISS	Shoe-Integrated Sensor System
ECG	Electrocardiography
EMG	Electromyography
EEG	Electroencephalography
SWCTNet	Sliding Window CNN + Channel-Time Attention Transformer Network
PPG	Photoplethysmography
3D	Three-Dimensional
FIR	Finite Impulse Response
IIR	Infinite Impulse Response
LMS	Least Mean Squares
STFT	Short-Time Fourier Transform
EMD	Empirical Mode Decomposition
RLE	Run-Length Encoding
DWT-RI	Discrete Wavelet Transform–Recursive Inverse
MRA	Multiresolution Analysis
MAs	Motion Artifacts
CNNs	Convolutional Neural Networks
COVID-19	Coronavirus Disease 2019
